# Does rural talent cultivation enhance farmers’ social capital? Evidence from the ‘One Village, One University Student’ project

**DOI:** 10.1371/journal.pone.0357662

**Published:** 2026-09-16

**Authors:** Jiawei Wang, Xia Kuang, Hui Yang, Wenmei Liao, Hailan Qiu

**Affiliations:** 1 School of Health Management, Nanchang Medical College, Nanchang, China; 2 School of Economics and Management, Jiangxi Agricultural University, Nanchang, China; Shanghai Ocean University, CHINA

## Abstract

Using the One Village, One University Student (OVOUS) project as an example and drawing on survey data from 2,552 farmers in 12 counties (county-level cities/districts) in Jiangxi Province, China, this study employs an endogenous switching probit (ESP) model to empirically examine whether participation in rural revitalization talent-training programs enhances farmers’ social capital. The results show that: (1) participation significantly increases farmers’ bonding social capital by 6.8 percentage points (ATT = 0.068, SE = 0.002), suggesting that systematic education not only strengthens individual capabilities but also facilitates the expansion of social networks; (2) correspondence education yields a stronger social-capital-promoting effect than distance education, with estimated effects of 8.1 and 5.8 percentage points, respectively, highlighting the potential role of face-to-face interaction and repeated social engagement in enhancing farmers’ bonding social capital; and (3) older farmers experience slightly larger gains in bonding social capital than younger farmers (10.8 versus 10.5 percentage points), and female farmers experience substantially larger gains than male farmers (12.8 versus 5.1 percentage points), indicating stronger marginal returns among groups with relatively weaker social networks. Overall, the findings reveal that rural talent policies generate important spillover value by fostering social capital accumulation. This implies that future policy design should jointly consider the dual effects on human capital and social capital and, in light of group heterogeneity, improve the matching mechanisms between training modalities and target participants to enhance policy precision and overall effectiveness.

## Introduction

The report of the 19th National Congress of the Communist Party of China put forward the “implementation of the strategy of rural revitalization” for the first time and pointed out that the revitalization of talent is critical. To promote rural revitalization, it is necessary to adhere to the main position of farmers and focus on enhancing their development potential. Since the reform and opening up, the surplus rural labor force has been moving to the cities on a large scale, especially young and middle-aged people, thus resulting in a shortage of human resources for the revitalization of the countryside. Therefore, a stable system of cultivating local expertise in the countryside is considered the cornerstone of stimulating the endogenous momentum of rural revitalization [1]. To alleviate the shortage of rural expertise and accelerate the modernization of agriculture and rural areas, the Ministry of Education implemented the One Village, One University Student (OVOUS) project in 2004, aiming to cultivate highly skilled individuals and grassroots administrators who meet the needs of rural development. This talent cultivation project adopts the model of “government funding, university contribution, farmer benefit,” bringing higher education resources into rural areas through adult education and directly benefitting farmers. This initiative has developed numerous rural practical talents, who are “rooted in the countryside, serving farmers, developing agriculture and leading the way to prosperity,” and has become crucial to promote the revitalization of rural expertise. In 2021, the General Office of the CPC Central Committee and the General Office of the State Council jointly issued the ‘Opinions on Accelerating the Revitalization of Rural Talents’, emphasizing the importance of implementing the OVOUS project to strengthen the construction of specialist echelons in rural areas. As a key project for rural revitalization talent cultivation, this project has been underway for several years, and an evaluation of its effectiveness has become a practical issue that urgently requires in-depth research.

Since the implementation of the OVOUS project, the academic community has paid close attention to it. There are abundant research results, mainly focusing on the rural talent cultivation mode [2], construction of the education and teaching quality evaluation system and current situation of talent cultivation and the problems faced [3,4]. Many scholars have recognized the significant effects of the project [5], which is mainly reflected in the enhancement of farmers’ willingness to professionalize [6] and engage in agricultural entrepreneurship [7]. Meanwhile, studies have concluded that the rural revitalization talent cultivation is the key to enhancing farmers’ social capital [8]. However, there is still a lack of empirical research to verify whether the project can enhance farmers’ social capital effectively. Social capital, which originated from sociological research and later became a key explanatory and descriptive factor in individual development and economic growth, represents the social resources of individuals in maintaining their livelihood, pursuing development and responding to risks, and its essence is the ability to obtain resources through social relationships established by trust, participation and prestige [9]. Researchers have focused on the impact of social capital on individual behavior, for example, in the ‘humanized’ environment of rural China, social capital improves not only agricultural productivity but also life satisfaction [10,11]. It can also regulate the relationship between land transfer and farmers’ entrepreneurship [12], promote the upgrading of household consumption and alleviate multidimensional poverty in the household [13]. However, there are relatively few studies on the path of social capital enhancement, and the influence of rural revitalization talent cultivation on it deserves in-depth exploration.

In summary, existing studies have examined the relationship between rural education and social capital from perspectives such as educational attainment, skills training, or social participation, yet several limitations remain. First, most studies focus on educational level or general training experiences, paying insufficient attention to policy-oriented rural revitalization talent-training programs and lacking a systematic examination of the relevant institutional context. For example, existing studies on the OVOUS project mainly examine talent-training modes, teaching quality, professionalization, and agricultural entrepreneurship [2–7], rather than its effects on farmers’ social capital. Second, much of the evidence is based on correlational analysis and rarely addresses farmers’ self-selection into training, which may lead to underestimation or overestimation of the true effects of training. Existing research linking rural talent cultivation to social capital has largely emphasized their theoretical or correlational relationship [8], without adequately accounting for farmers’ non-random participation in training. Third, the literature has not sufficiently distinguished between different training modalities and their mechanisms—particularly lacking comparative analyses of the differentiated impacts of correspondence education versus distance education. Previous studies on the OVOUS project generally evaluate its overall effects [5–7], with limited attention to differences between specific training modalities. Against this background, this study takes participants in the OVOUS project as the research subject and, from a micro-level perspective, systematically investigates the effects of rural revitalization talent-training programs on farmers’ social capital, while further analyzing heterogeneity across training modalities. Using survey data from 2,552 farmers in 12 counties (county-level cities/districts) in Jiangxi Province, this study applies an endogenous switching probit (ESP) model and a counterfactual analysis framework to identify the causal effect of farmers’ participation in rural revitalization talent-training programs and to compare differences in social-capital-promoting effects between correspondence education and distance education.

The marginal contributions of this study are threefold. First, grounded in the context of the rural revitalization strategy, it systematically evaluates the impact of farmers’ participation in rural revitalization talent-training programs on social capital, thereby extending the policy context of research on rural education and social capital. Unlike previous studies that mainly focus on professionalization and agricultural entrepreneurship [6,7], this study examines the social-capital effects of rural talent cultivation. Second, by employing an endogenous switching probit (ESP) model to identify causal effects, it accounts for potential sample self-selection bias. Unlike existing research that primarily discusses the relationship between rural talent cultivation and social capital without explicitly addressing self-selection [8], this study uses the ESP model and counterfactual analysis to account for both observed and unobserved selection factors. Third, by further examining training modalities and individual characteristics, it reveals heterogeneous patterns of social capital improvement across different groups and different training approaches, providing empirical evidence to support the optimization of rural revitalization talent-training policies.

## Theoretical research hypotheses and design of variables

### Theoretical analysis of the effects of rural revitalization talent-training programs on farmers’ social capital

Rural revitalization talent-training programs not only bear the direct mission of enhancing farmers’ human capital [14], but also exert profound influences on rural social networks and the configuration of social capital by reshaping patterns of social interaction and resource allocation. Drawing on the core dimensions of social capital theory, this study develops an analytical framework of “network restructuring–resource empowerment–norm reconfiguration” to systematically elucidate the mechanisms through which farmers’ participation in rural revitalization talent-training programs promotes social capital accumulation. Specifically, this study mainly follows Bourdieu’s perspective that social capital represents the actual or potential resources embedded in social relationships, emphasizing individuals’ ability to obtain resources through established networks [13,15]. Coleman’s view on institutional embeddedness and Putnam’s emphasis on trust and norms are further incorporated to explain the mechanisms through which training programs reshape resource access and interpersonal relationships. It should be noted that the social capital indicator used in this study—whether a farmer can borrow a certain amount of money from relatives or friends—primarily captures farmers’ capacity to access resources through kinship- and locality-based ties, thereby reflecting bonding social capital. While this measure effectively reflects mutual assistance and support within acquaintance-based networks, it cannot directly capture bridging social capital, which is characterized by connections across different social groups.

First, skill acquisition and social network restructuring. Bourdieu defines social capital as the aggregate of actual or potential resources linked to possession of a durable network of institutionalized social relationships at the individual or group level [13,15]. Rural revitalization talent-training programs, through systematic skills training and organized, intensive learning arrangements, create structural opportunities for farmers to expand the boundaries of their social interactions. During training, farmers participate in centralized instruction, technical exchanges, and experience-sharing activities, gradually moving beyond relatively closed networks dominated by kinship and locality to establish cross-group and cross-hierarchical connections with heterogeneous actors such as agricultural experts, business operators, and cooperative leaders [15]. This expansion of networks characterized by weak ties not only increases the diversity of farmers’ social relations, but also substantially enhances their capacity to obtain heterogeneous information and external resources, thereby laying a network foundation for social capital accumulation.

Second, institutional embeddedness and structural resource empowerment. Coleman emphasizes the structural nature of social capital and argues that its sustained accumulation depends on support from institutional arrangements [16]. Rural revitalization talent-training programs, through institutionalized design, embed farmers into new channels for resource access and public participation. At the policy level, the establishment of an integrated institutional system linking “education and training–qualification certification–policy support” enables participating farmers to obtain more development opportunities within formal institutional frameworks. On the one hand, farmers who obtain relevant certifications are granted priority in credit support, project applications, and policy coordination; on the other hand, talent certification functions as an institutionalized signal that enhances farmers’ credibility and visibility in market transactions and public affairs. Such institutional embeddedness not only empowers farmers in terms of resources, but also strengthens their capacity to participate in grassroots governance and public affairs, thereby expanding the institutional basis of their social capital.

Third, identity recognition and the reconfiguration of trust norms (knowledge diffusion and normative mechanisms). Putnam points out that social capital is reflected not only in network structures but also in cultural and cognitive dimensions such as trust and norms [17]. By strengthening knowledge diffusion and professional competence recognition, rural revitalization talent-training programs promote the reconstruction of farmers’ occupational identity. When farmers acquire new occupational identities—such as “rural artisans” or “local experts”—through systematic training, their professional knowledge and technical capabilities gain recognition within the community. This not only enhances individuals’ self-efficacy but also fosters, at the community level, a trust mechanism grounded in professional competence. Compared with the acquaintance-based trust typical of traditional relationship-oriented societies, this competence-based trust is more open and scalable. It helps overcome the constraints of limited trust, facilitates the formation of norms for knowledge sharing and cooperation, and substantially increases farmers’ voice in community governance and collective decision-making.

In summary, rural revitalization talent-training programs systematically promote the accumulation and upgrading of farmers’ social capital through multiple mechanisms, including social network expansion, institutional resource empowerment, and the reconfiguration of trust and norms. Among these mechanisms, the improvement of farmers’ ability to mobilize resources through existing social networks is directly reflected in the measurement of bonding social capital adopted in this study, whereas network expansion, institutional support, and trust formation provide potential explanations for this outcome. This process not only provides sustained empowerment for farmers’ individual development but also lays a solid social foundation for optimizing rural social structures and advancing the rural revitalization strategy in the long run. Based on the above analysis, this study proposes the following hypothesis:

H1: Participation in rural talent revitalization training programs enhances farmers’ bonding social capital.

### Theoretical analysis of how different training modalities affect farmers’ social capital

Within the implementation of the rural revitalization strategy, differences between correspondence education and distance education in their effects on improving farmers’ social capital essentially stem from the distinct forms of social interaction and relationship-building mechanisms shaped by different training media. The formation of social capital depends not only on acquiring knowledge and skills, but also—crucially—on the gradual accumulation of trust, social identification, and stable social networks through sustained interaction. It is therefore necessary to systematically analyze how different training modalities shape farmers’ social capital formation mechanisms from the perspectives of interaction patterns, social learning processes, and social network structures.

First, differences in interaction depth shape the accumulation of relational social capital. According to social presence theory [18], correspondence education, through periodic, face-to-face intensive learning and communication, creates interaction settings with high social presence and high frequency, which facilitates multidimensional social learning among participants. Such face-to-face interaction not only promotes the transfer of explicit knowledge, but also cultivates shared cognition and professional identification through observational learning and experience exchange [19], making it easier for participants to form strong relational ties based on trust and reciprocity [20,21]. By contrast, distance education, due to its asynchronous nature and spatial dispersion, filters out social cues to some extent; interaction among participants tends to take the form of low-frequency and fragmented information exchange, thereby constraining the formation of group belonging and trust. Because social capital relies heavily on trust, norms of reciprocity, and emotional bonds formed through repeated interactions, the face-to-face communication and high-frequency interaction environment provided by correspondence education is more conducive to building stable and enduring relational social capital among farmers, whereas distance education is relatively limited in fostering deep, cumulative trust.

Second, differences in social network structure affect pathways of resource access and social capital conversion. Correspondence education typically organizes participants into fixed classes, within which members maintain stable contact over an extended period, gradually forming a high-density, strongly connected, and relatively closed network structure. In such networks, members often assume multiple roles simultaneously—such as technical exchange, information sharing, and mutual assistance—while repeated interactions strengthen informal constraints and improve the efficiency of mobilizing internal resources. In contrast, distance education more readily forms an open network structure centered on platforms or instructors. Relationships among participants are more likely to be weak ties; although this may facilitate information diffusion, the capacity for resource integration and sustained mutual assistance is relatively limited [22]. Especially in rural contexts, whether social networks can be effectively converted into social capital often depends on the stability and continuity of network relations. Accordingly, the high-density networks formed through long-term offline contact in correspondence education are more likely to translate into sustained resource mutual aid and social support, whereas the weak-tie networks formed through distance education may face constraints in their efficiency of social capital conversion due to real-world conditions [23].

Third, differences in the degree of institutional embeddedness influence social identity recognition and the formation of reputational capital. Rural revitalization talent training is not only a process of transmitting knowledge and skills, but also an important carrier of institutional empowerment. Correspondence education is often more closely linked institutionally with local governments, universities, and grassroots organizations, and its training process and outcomes are frequently accompanied by clear academic credentials or qualification certifications. Such institutional embeddedness helps strengthen farmers’ identity recognition within village society and grassroots governance systems, making them more likely to be regarded as key actors who are “technically skilled and capable,” thereby enhancing their social reputation and social recognition [24]. In comparison, distance education relies more on platform-based and technology-mediated approaches; the visibility of training outcomes and the degree of institutional recognition in grassroots communities are relatively weaker, meaning that its social-capital effects depend to a greater extent on individual differences in capability.

Based on the above analysis, given its higher interaction intensity, more stable social network structure, and stronger institutional embeddedness, correspondence education is expected to generate more pronounced effects on social capital improvement than distance education. Accordingly, this study proposes the following hypothesis:

H2: Correspondence education in rural revitalization talent-training programs increases farmers’ bonding social capital by a larger margin than distance education.

## Data, variables and methods

### Data sources

Jiangxi Province is one of China’s major agricultural provinces and a key rice-producing region. However, for a long time, the stock of rural human capital has been relatively insufficient, and the overall quality of the labor force has remained low, making it difficult to meet the practical demand of the rural revitalization strategy for high-quality, versatile rural talent. Against this backdrop, Jiangxi Province took the lead in launching the OVOUS project in 2012, with the aim of systematically cultivating high-level and high-quality practical rural talent. The project combines *correspondence education* with *distance education* and adopts diversified teaching approaches—including on-campus study, a flexible schooling system, alternating between agricultural work and study, and delivering instruction to rural areas—to provide higher vocational education for key rural groups such as specialized large-scale farming households, heads of leading agribusiness enterprises, leaders of cooperative organizations, and members and reserve cadres of village-level “two committees.” Existing studies show that the OVOUS project has significantly strengthened participating farmers’ intentions toward entrepreneurship and remaining in agriculture, playing a positive role in promoting industrial revitalization and increasing farmers’ income [25].

With the deepening of the rural revitalization strategy, Jiangxi Province issued the *Implementation Opinions on Advancing the One Village, One University Student Project in Depth* in 2020. Building on earlier efforts, the province continued to expand the scope of trainees by incorporating leaders of rural social service organizations, returning migrant workers, and returning entrepreneurial youth into the training system, and gradually extended the training level from junior college and undergraduate programs to postgraduate programs. To date, the OVOUS project has been implemented continuously in Jiangxi for 12 years, enrolling more than 70,000 farmer participants. As a result, the number of farmers with higher education increased from an average of fewer than 0.4 per administrative village in 2012 to nearly 5. In terms of duration, scale, training level, and coverage, Jiangxi ranks among the leading provinces nationwide, making it highly demonstrative and representative. Therefore, selecting Jiangxi as the study area helps examine the implementation effects of rural revitalization talent training within a relatively mature and systematic policy context.

Regarding data collection, the research team conducted a household survey in December 2019. The sample included farmers who participated in the OVOUS project after 2012 as the treatment group, and farmers who did not participate as the control group. To enhance representativeness, the survey adopted a multi-stage stratified random sampling strategy. First, 12 counties (county-level cities/districts) were randomly selected from Jiangxi Province based on per capita industrial value added. Second, using per capita public fiscal revenue as the stratification criterion, three townships were randomly selected from each county. Third, three administrative villages were randomly selected from each township. Finally, ten farmers who had not participated in the OVOUS project were randomly selected from each administrative village for interviews. In total, 2,805 questionnaires were distributed; after data cleaning and screening, 2,552 valid questionnaires were obtained, yielding an effective response rate of 90.98%. This sampling design accounted for within-province heterogeneity in geography, economic development, and individual farmer characteristics, thereby providing a relatively reliable data foundation for the empirical analysis.

### Ethics statement

The study protocol was approved by the Institutional Review Board of the Research Office of Jiangxi Agricultural University (Approval No. JXAULL-2018032; approved on December 24, 2018). Written informed consent was obtained from all participants before data collection. Before participation, members of the research team provided each participant with written information describing the purpose and procedures of the study, the voluntary nature of participation, confidentiality protections, the use of anonymized data for academic research, and the right to withdraw at any time without adverse consequences. All participants consented to the use, analysis, and publication of their anonymized data for academic purposes.

### Selection of variables

1. Explained variable: Farmers’ bonding social capital is selected. This study refers to existing research [26,27] and divides social capital into two categories, namely, bonding social capital, referring to close social relationships formed through blood ties, ethnicity and family ties, and bridging social capital, referring to social relationships connected through colleagues and friends. In this study, the measurement of social capital focuses specifically on bonding social capital, as the available indicator reflects farmers’ ability to mobilize resources through close interpersonal networks rather than broader connections across social groups. Accordingly, the question ‘If there is an emergency at home, can you get a 5,000-yuan loan from friends and family?’ is used as a measure of farmers’ capacity to access reciprocal financial support within their bonding social networks.
2. Key explanatory variable: It involves whether farmers participate in rural revitalization talent cultivation. In this study, whether farmers participate in the OVOUS project is used as a proxy variable for the cultivation of talent in rural revitalization, focusing on the impact of participating in the OVOUS project on the enhancement of farmers’ social capital. Notably, there may be a reverse causality problem between the causal variables. On the one hand, participation in the OVOUS project may promote the enhancement of farmers’ social capital; on the other hand, farmers with rich social capital tend to be more likely to participate in the OVOUS project. Therefore, measures will be taken in the empirical analysis to address the resulting endogeneity problem.
3. Control variables: Based on existing studies [28], the following control variables were selected. First, individual control variables, including age, gender, whether they are party members and whether they are engaged in non-agricultural work. Second, household characteristics control variables, including the number of family members, actual area of arable land operated and number of people in the labor force. Third, locational characteristics control variables, including the topography of the villages, distance from villages to the county towns and economic development level of the region.
4. Instrumental variable: farmers’ knowledge of the OVOUS project policy. To address potential reverse causality and ensure model identification, this study introduces farmers’ awareness of the OVOUS policy as an instrumental variable. This variable is suitable as an instrument primarily because it operates by influencing farmers’ decision-making regarding whether to participate in rural revitalization talent-training programs. After controlling for potential confounders such as individual characteristics, household characteristics, and locational factors, policy awareness itself neither directly alters farmers’ existing social relationship structures nor significantly increases the stock of mobilizable resources within their social networks. Any potential impact on social capital is mainly realized indirectly through the mediating pathway of “participation in rural revitalization talent training.” Therefore, in theoretical terms, this instrument satisfies the exclusion restriction, i.e., it does not directly affect the dependent variable, and thus meets the conditions for a valid instrumental variable. Detailed variable definitions and descriptive statistics are reported in Table 1.

**Table 1:** Descriptive analysis of variables.

Variable name	Variable definition	Mean	SD
1. Explained variables			
Social capital	Can you get a loan from friends and relatives in the event of a family emergency? Yes = 1, No = 0	0.835	0.372
2. Explanatory variables			
Rural revitalization talent cultivation	Are you a participant in the OVOUS project? Yes = 1, No = 0	0.589	0.492
3. Individual characteristics			
Age	Age of respondent in years	46.971	12.779
Gender	Male = 1, Female = 0	0.757	0.429
Political party member	Yes = 1, No = 0	0.458	0.498
Engaged in non-agricultural work	Yes = 1, No = 0	0.650	0.477
4. Family characteristics			
Number of family members	Total number of actual household members	4.796	1.802
Number of foreign workers	Number of family members working outside the home	1.472	1.381
Number of laborers	Number of family laborers	2.762	1.348
Cropland area	Actual area of cultivated land in operation, acres	9.248	49.181
5. Location characteristics			
Village topography	Plains = 1, Hills = 2, Mountains = 3	2.222	0.729
Distance from villages to county towns	Distance from village to county town, kilometers	27.105	20.624
Level of regional economic development	Disposable income per capita, logarithmic	9.582	0.255
6. Instrumental variables			
Level of policy understanding	Knowledge of the OVOUS project policy? 1 = no knowledge; 2 = some knowledge; 3 = good knowledge	1.989	0.978

### Descriptive statistics

The mean and standard deviation of the variables for the whole sample is provided in Table 1. Overall, the sample presents the following characteristics. First, 1502 farmers in the sample participated in rural revitalization talent cultivation, accounting for 58.9% of the sample, of which 809 were in correspondence education and 693 were in distance education. Moreover, 1,050 were non-participants, accounting for 41.1% of the sample. The mean value of social capital was 0.835, indicating that most farmers could get support from friends and relatives by borrowing 5,000 yuan in an emergency. Furthermore, the participation in non-farm work is as high as 0.650, emphasizing its importance as the main source of household income. In addition, the average age of the sample was 46.971 years, with a gender mean of 0.757, reflecting that the respondents were predominantly middle-aged and older males and had a higher proportion of party members (with a mean of 0.458). Notably, despite the high proportion of farmers participating in rural revitalization talent cultivation, the mean value of their understanding of the policy is only 1.989, indicating that the sample’s overall awareness of the policy needs to be improved.

Table 2 further compares the differences in the means of the variables for the ‘participant’ and ‘non-participant’ groups and the two modes of correspondence education and distance education. The results show that participants in the OVOUS project are younger, have a higher proportion of party members, have a larger area of family farmland and have more migrant workers than non-participants. Both correspondence education and distance education can enhance the social capital of farmers, and the difference is significant at the 1% level, which preliminarily verifies the positive effect of the two training methods. However, the difference between the mean values of social capital, although existing, is not sufficient to be attributed directly to participation in rural revitalization talent cultivation. Therefore, to assess the impact of participation in rural revitalization talent cultivation on farmers’ social capital accurately, a more rigorous econometric analysis method is needed for in-depth exploration.

**Table 2:** Differences in the means of variables for participation in rural revitalization talent cultivation and different training methods.

Variable name	Participation		Training method		Variance I	Variance II	Variance III
	Yes	No	Correspondence	Distance			
1. Explained Variables							
Social capital	0.917	0.717	0.916	0.917	0.197^***^	0.194^***^	0.210^***^
	(0.276)	(0.451)	(0.277)	(0.245)			
2. Explanatory variables							
Rural revitalization talent cultivation	1	0	1	1	1	1	1
	(0.000)	(0.000)	(0.000)	(0.000)			
3. Individual farmer characteristics							
Age	39.363	57.871	38.936	39.860	−18.508^***^	−18.935^***^	−18.011^***^
	(7.390)	(10.829)	(7.536)	(7.190)			
Gender	0.725	0.804	0.719	0.731	−0.079^***^	−0.085	−0.073
	(0.447)	(0.397)	(0.449)	(0.444)			
Political party member	0.651	0.182	0.682	0.614	0.469^***^	0.500^***^	0.432^***^
	(0.477)	(0.386)	(0.466)	(0.487)			
Engaged in non-agricultural work	0.859	0.350	0.868	0.850	0.509^***^	0.518^***^	0.500^***^
	(0.438)	(0.477)	(0.339)	(0.357)			
4. Family characteristics							
Number of family members	5.024	4.469	4.993	5.061	0.555^***^	0.524^***^	0.592^***^
	(1.623)	(1.986)	(1.601)	(1.648)			
Number of foreign workers	0.831	1.919	1.985	1.843	1.109^***^	0.909^***^	0.591^***^
	(1.113)	(1.373)	(1.436)	(1.293)			
Number of laborers	3.053	2.345	3.057	3.048	0.708^***^	0.712^***^	0.703^***^
	(1.240)	(1.386)	(1.280)	(1.194)			
Cropland area	12.413	4.713	12.974	11.931	7.700^***^	7.218^***^	8.261^***^
	(62.754)	(14.450)	(66.728)	(59.170)			
5. Location Characteristics							
Village topography	1.966	2.589	1.991	1.935	−0.623^***^	−0.598^***^	−0.654^**^
	(0.723)	(0.561)	(0.717)	(0.731)			
Distance from villages to county towns	25.102	29.974	25.507	24.629	−4.872^***^	−4.467^***^	−5.345^***^
	(19.643)	(21.642)	(20.284)	(18.872)			
Level of regional economic development	9.586	9.576	9.584	9.589	0.010	0.004	0.004
	(0.239)	(0.205)	(0.238)	(0.241)			
6. Instrumental variables							
Level of policy understanding	2.428	1.359	2.719	2.089	1.069^***^	1.360^***^	0.730^***^
	(0.891)	(0.717)	(0.874)	(0.784)			
N	1502	1050	809	693			

**Notes:** 1) ***, ** and * indicate significance at the 1%, 5% and 10% levels, respectively. The number in brackets is the standard error. The same applies to the following table.; 2) Difference 1 is the result of comparing farmers who participated and did not participate in rural revitalization talent cultivation, difference 2 is the result of comparing farmers who participated in rural revitalization talent cultivation correspondence education and those who did not, difference 3 is the result of comparing farmers who participated in rural revitalization talent training through distance education.

### Measurement methods

In theory, farmers’ participation in rural revitalization talent-training programs can enhance their social capital; however, the level of social capital may also shape farmers’ willingness to participate, which can give rise to reverse causality and self-selection bias. Although this study controls for a wide range of factors, it remains difficult to fully rule out interference from unobserved variables. While propensity score matching can mitigate differences in observable characteristics, it cannot adequately correct biases driven by latent factors. Therefore, following Lokshin [29,30], this study employs an endogenous switching probit (ESP) model to jointly estimate the participation decision and the outcome equation, thereby identifying the causal effect of training in a more robust manner.

The ESP model simultaneously estimates the following equations:


$$\begin{array}{c} Y_{i}=\beta^{\prime }X_{i}+\delta^{\prime }T_{i}+\varepsilon_{i}Y_{i}=\beta^{\prime }X_{i}+\delta^{\prime }T_{i}+\varepsilon_{i} \end{array}$$ (1)

(1) In the equation, $Y_{i}$ denotes the level of farmers’ social capital; $X_{i}$ is the individual characteristics, family characteristics, location characteristics and other factors that affect the level of farmers’ social capital; $T_{i}$ is the decision term of whether farmers participate in rural revitalization talent cultivation; $T_{\text{1}}$ indicates that farmers participate in rural revitalization talent cultivation and $T_{\text{0}}$ indicates that farmers do not participate in rural revitalization talent cultivation. In addition, $\beta^{\prime }$ ^and^ $\delta^{\prime }$ are the coefficients to be estimated; $\varepsilon_{i}$ is the random error term.

The ESP model consists of two stages. In the first stage, a probit model is used to estimate the determinants of farmers’ participation in the talent-training program as well as the choice between different training modalities. In the second stage, the outcome equation is estimated to evaluate the effects of training participation and training modality on farmers’ social capital. Specifically, the model is specified as follows:

### Phase I

Behavioral equations (whether farmers participate in rural revitalization talent cultivation):


$$\begin{array}{c} T_{i}=\gamma Z_{i}+\mu_{i}T_{i}=\gamma Z_{i}+\mu_{i} \end{array}$$ (2)

Behavioral equations (whether farmers participate in correspondence education for rural revitalization talent cultivation):


$$\begin{array}{c} T_{i^{\prime }}=\gamma Z_{i^{\prime }}+\mu_{i^{\prime }}T_{i^{\prime }}=\gamma Z_{i^{\prime }}+\mu_{i^{\prime }} \end{array}$$ (3)

### Phase II

Resulting equation 1 (treatment group 1, farmers’ level of social capital to participate in rural revitalization talent cultivation):


$$\begin{array}{c} Y_{\text{1}}=\eta_{\text{1}}X_{i}+{\nu_{\text{1}}}_{i} \end{array}$$ (4-a)

Resulting equation 2 (treatment group 2, level of social capital of farmers participating in rural revitalization talent cultivation correspondence education):


$$\begin{array}{c} Y_{\text{2}}=\eta_{\text{2}}X_{i}+\nu_{\text{2}i} \end{array}$$ (4-b)

Resulting equation 3 (treatment group 3, level of social capital of farmers participating in rural revitalization talent cultivation distance education):


$$\begin{array}{c} Y_{\text{3}}=\eta_{\text{3}}X_{i}+\nu_{\text{3}i} \end{array}$$ (4-c)

Resulting equation 4 (treatment group 4, level of social capital of farmers not participating in rural revitalization talent cultivation):


$$\begin{array}{c} Y_{\text{4}}=\eta_{\text{4}}X_{i}+\nu_{\text{4}i} \end{array}$$ (4-d)

In equations (2) and (3), $T_{i}$ is a binary choice variable indicating whether farmers participate in rural revitalization talent cultivation; $T_{i^{,}}$ indicates whether farmers participate in rural revitalization talent cultivation correspondence education; $Z_{i}$ is a series of factors influencing whether farmers participate in rural revitalization talent cultivation; $Z_{i^{\prime }}$ is a series of factors affecting whether farmers participate in rural revitalization talent cultivation; $\mu_{i}$ is an error term, representing unobservable influences, such as whether the personality of the ‘principal’ of the sample farmer’s family is extroverted, and labor habits.

In equations (4-a), (4-b), (4-c) and (4-d), $Y_{\text{1}}$,$Y_{\text{2}}$,$Y_{\text{3}}$ and $Y_{\text{4}}$ denote the level of social capital of farmers participating in rural revitalization talent cultivation, participating in rural revitalization talent cultivation by correspondence education, participating in rural revitalization talent cultivation by distance education and not participating in rural revitalization talent cultivation, respectively. Furthermore, $\eta_{\text{1}}$, $\eta_{\text{2}}$, $\eta_{\text{3}}$ and $\eta_{\text{4}}$ denote the parameters to be estimated in each equation, respectively; $X_{i}$ is a series of factors affecting farmers’ social capital and $\nu_{\text{1}i}$*,* $\nu_{\text{2}i}$*,* $\nu_{\text{3}i}$ *and* $\nu_{\text{4}i}$ form the error term in the resulting equation.

The ESP estimation results reveal heterogeneous effects of various factors on social capital for farmers who participated in training versus those who did not. To evaluate the overall effect of training, it is necessary to compute the average treatment effect on the treated (ATT) on social capital based on the estimated model coefficients. The calculation is as follows:


$$\begin{array}{c} ATT=E\left(Y_{i}\right|T_{i}=\text{1})-E\left(Y_{i}\right|{-T}_{i}=\text{0}) \end{array}$$ (5)

$$\begin{array}{c} ATT=E\left(Y_{i^{\prime }}|T_{i^{\prime }}=\text{1}\right)-E(Y_{i^{\prime }}|T_{i^{\prime }}=\text{0}) \end{array}$$ (6)

In equations (5) and (6), $E\left(Y_{i}\right|T_{i}=\text{1})$ denotes the expected average social capital if all sample farmers participate in rural revitalization talent cultivation; $E\left(Y_{i^{\prime }}|T_{i^{\prime }}=\text{1}\right)$ denotes the expected average social capital if all sample farmers participate in rural revitalization talent cultivation correspondence education; $E\left(Y_{i}\right|T_{i}=\text{0})$ is the expected average social capital if all sample farmers did not participate in rural revitalization talent cultivation.

In the empirical estimation, this study applies full information maximum likelihood (FIML) to jointly estimate the selection equation and the outcome equation. Specifically, the ESP model is estimated using the user-written switch_probit command in Stata 18, which implements a full-information maximum likelihood estimator for endogenous switching probit models. This approach allows the error term in the selection equation to be correlated with the error term in the outcome equation, thereby accounting for potential self-selection associated with unobserved factors. The model tests for the presence of selection bias by estimating the correlation coefficient (ρ) between the error terms: if ρ is statistically significant and differs from zero, this indicates correlation between the selection and outcome equations, implying that estimation using the ESP model is necessary and appropriate; conversely, if ρ is not statistically significant, selection bias is not pronounced.

## Model estimation results and analysis

### ESP model estimation results and analysis

Table 3 reports the estimation results of the ESP model of the impact of farmers’ participation in rural revitalization talent cultivation on their social capital. The Wald test rejects the original hypothesis that the behavioral and outcome equations are independent of each other at the 1% level of significance, indicating that the model possesses strong significance overall. The estimated value of rho0 is statistically significant at the 10% level, suggesting a significant correlation between the error terms of the selection and outcome equations. This implies the presence of sample self-selection bias, making joint estimation using the endogenous switching probit (ESP) model necessary. This finding also provides structural support for the role of the instrumental variable in identifying the participation decision.

**Table 3:** Estimated results of the impact of participation in rural revitalization talent cultivation on farmers’ bonding social capital.

Variable name	Rural revitalization talent cultivation	Farmers’ bonding social capital			
		Participated	Non-participation	Correspondence	Distance
1. Individual farmer characteristics					
Age	−0.011^***^	−0.011	−0.019^***^	−0.011	−0.019^***^
	(0.001)	(0.009)	(0.005)	(0.012)	(0.005)
Gender	−0.016	0.015	0.200^*^	0.009	−0.005
	(0.010)	(0.112)	(0.110)	(0.158)	(0.614)
Political party member	0.188^***^	0.159	0.230^*^	0.190	0.104
	(0.014)	(0.113)	(0.122)	(0.182)	(0.222)
Engaged in non-agricultural work	0.059^***^	0.409^***^	−0.072	0.556^***^	0.212
	(0.013)	(0.127)	(0.100)	(0.170)	(0.202)
2. Family characteristics					
Number of family members	0.003	−0.042	0.017	−0.031	−0.049
	(0.003)	(0.035)	(0.043)	(0.038)	(0.048)
Number of laborers	−0.005	−0.021	0.012	−0.028	−0.016
	(0.005)	(0.057)	(0.061)	(0.079)	(0.085)
Number of foreign workers	0.031^***^	−0.001	−0.144^***^	0.008	−0.019
	(0.004)	(0.045)	(0.051)	(0.063)	(0.071)
Cropland area	0.001^***^	0.001	0.006	0.001	0.001
	(0.000)	(0.001)	(0.003)	(0.001)	(0.001)
3. Location characteristics					
Village topography	−0.066^***^	0.038	−0.076	0.013	0.016
	(0.006)	(0.059)	(0.079)	(0.099)	(0.121)
Level of regional economic development	0.049^***^	0.416^**^	−0.824^***^	0.507^*^	0.285
	(0.019)	(0.198)	(0.213)	(0.275)	(0.292)
Distance from villages to county towns	−0.000^**^	0.001	−0.001	0.001	0.001
	(0.000)	(0.002)	(0.002)	(0.003)	(0.003)
4. Instrumental variables					
Level of policy understanding	0.047^***^				
	(0.005)				
constant term (math.)		−2.390	9.456^***^	−3.489	−0.896
		(1.939)	(2.058)	(2.683)	(2.868)
athrho1/ athrho0		0.273	0.255^*^		
		(0.211)	(0.148)		
rho1/ rho0		0.266	0.249^*^		
		(0.195)	(0.139)		
independence test		Wald chi2(12)=605.25^***^ chi2(2)=4.72 Prob>chi2 = 0.0944			
N	2552	1502	1050	809	693

**Notes:** Standard errors are in parentheses. The added average marginal effects refer only to the participation/selection equation and are computed from the jointly estimated ESP model using predict(psel). For continuous variables, entries are sample-average derivatives. For gender, party membership, and non-agricultural work, entries are average discrete changes from 0 to 1. No marginal effect is defined for the constant, correlation parameters, or independence test. * p < 0.10, ** p < 0.05, *** p < 0.01.

Within the ESP framework, the validity of the instrumental variable depends on whether it satisfies two fundamental conditions: relevance and exogeneity. Regarding relevance, the selection-equation estimates show that policy awareness has a statistically significant effect on farmers’ participation in rural revitalization talent training, indicating a robust statistical association between the instrument and the endogenous explanatory variable and thus meeting the relevance requirement. Regarding exogeneity, after controlling for potential confounders such as individual characteristics, household characteristics, and locational factors, policy awareness does not directly affect farmers’ existing social relationship structures or the stock of mobilizable resources within their social networks. Its influence on social capital is primarily realized indirectly through the mediating channel of the participation decision. Therefore, this variable is theoretically expected to satisfy the exclusion restriction. Taken together, the results support using policy awareness as an instrumental variable in the ESP model, which helps improve identification validity and enhances the credibility of the estimated effects.

Column 1 of Table 3 reports the average marginal effects from the selection equation estimating the determinants of farmers’ participation in rural revitalization talent cultivation. In terms of individual characteristics, farmers who are female, younger and party members are more likely to participate. The possible reasons for this are women’s lower education level and limited off-farm employment opportunities; furthermore, education positively contributes to a higher female labor supply and household income. Therefore, this group expects to strengthen agricultural production and income by participating in rural revitalization talent cultivation. In terms of family and location characteristics, the number of migrant workers, cultivated land area of the family and regional economic development level all significantly and positively affect farmers’ participation in rural revitalization talent cultivation. The number of migrant workers, as the human capital of the family, influences farmers’ behavioral decisions [31]. The larger the area of family farmland, the more farmers tend to learn advanced technology to reduce production risks and stabilize income [32]. Therefore, farmers with abundant labor and a large area of arable land are more inclined to participate in rural revitalization talent cultivation.

### Estimation results and analysis of the ESP model of the factors affecting farmers’ social capital

Columns 3 and 4 in Table 3 report the estimation results of the factors affecting the social capital enhancement of farmers who participated and did not participate in rural revitalization talent cultivation, respectively. First, the actual cultivated area significantly and positively affects the social capital of both types of farmers. This finding is consistent with existing studies that show that an increase in cultivated land area is accompanied by social capital enhancement [33]. Second, the distance from the village to the county town significantly and positively affects the social capital of both types of farmers. Furthermore, older men significantly affect social capital enhancement after participating in rural revitalization talent cultivation. This is consistent with Christoforou’s finding that age positively affects social capital [34], as older groups have more leisure time to socialize and tend to cooperate.

### Results and analysis of average treatment effect estimates

Line 2 of Table 4 presents the estimation of the average treatment effect of participating in rural revitalization talent cultivation on farmers’ social capital (ATT = 0.068), which is statistically significant at the 1% level of significance. This indicates that participation in rural revitalization talent training increases farmers’ probability of obtaining emergency financial support from relatives and friends by 6.8 percentage points compared with the counterfactual scenario of non-participation. As a result, hypothesis H1 of this paper is verified, i.e., participation in rural revitalization talent cultivation has a significant positive effect on farmers’ social capital. The underlying reason may be that participation in rural revitalization talent training, by providing a systematic learning and interaction platform, enhances frequent interaction and information sharing among farmers, thereby facilitating the formation of trust-based relationships and norms of reciprocity and further promoting the accumulation of social capital. Related studies indicate that education and training not only improve individual capabilities, but also significantly foster the growth of social capital by expanding social networks, strengthening social participation, and raising levels of trust [35]. This mechanism is particularly critical in rural settings, where standardized training can effectively compensate for deficiencies in network resources and information access arising from dispersed social ties.

**Table 4:** Mean treatment effect of the impact of participation in rural revitalization talent cultivation on farmers’ bonding social capital.

	Sample size	ATT	SE	T-value
Average treatment effect of participation in rural revitalization talent cultivation	1502	0.068^***^	0.002	28.333
Average treatment effect of correspondence education	809	0.081^***^	0.004	21.733
Average processing effect of distance education	693	0.058^***^	0.003	18.041

Further, the third and fourth rows of Table 4 report the average treatment effect estimates of participation in rural revitalization talent cultivation correspondence education and distance education on farmers’ social capital, respectively (ATT of 0.081 and 0.058, respectively), both of which are statistically significant at the 1% level of significance. After controlling for observable and unobservable factors, although both modes of training have a significant positive effect on farmers’ social capital, the social capital effect of correspondence education is more prominent compared to distance education (8.1% > 5.8%). Therefore, hypothesis H2 was tested. The reason why correspondence education outperforms distance education in enhancing social capital may lie in its more frequent and higher–social presence face-to-face interactions, which facilitate repeated interaction, peer communication, and the formation of stable social networks during the learning process. Prior research suggests that learning environments characterized by face-to-face contact or high social presence are more conducive to deep interaction, the formation of trust, and the sustained development of social networks—mechanisms that are central to social capital accumulation [36]. By contrast, the fragmented interactions and weakened connectivity typical of distance education may constrain its ability to effectively translate social relationships into social capital.

### Heterogeneity analysis

Given that demographic characteristics may shape individuals’ access to social resources and interaction opportunities, this study further conducts exploratory heterogeneity analyses by age and gender to examine whether the effects of talent training vary across different groups.

To analyses the relationship between farmers’ participation in rural revitalization talent cultivation and the growth of their social capital in depth, this paper divides farmers into an older age group (≥47 years old) and a younger age group (<47 years old) based on whether their age exceeds the mean value of 47 years and then explores the differential impact of age heterogeneity on the enhancement of their social capital after participating in rural revitalization talent cultivation. The estimation results are reported in Table 5, which shows that participating in rural revitalization talent cultivation has a significant positive impact on their social capital in both the senior and junior age groups. Specifically, the probability of social capital enhancement is 10.8% and 10.5% higher for the senior and junior groups, respectively, compared to farmers who did not participate in training for expertise in rural revitalization. This suggests that participation in rural revitalization talent cultivation can promote the accumulation of social capital among farmers of different age groups significantly. However, in comparison, the increase in social capital gained by farmers in the senior age group through participation in rural revitalization talent cultivation is slightly higher than that of the junior age group, which is consistent with existing research that older farmers have accumulated richer social capital than young and middle-aged farmers [37].

**Table 5:** Comparison of the social capital effects of participation in rural revitalization talent training across age groups.

	Sample size	ATT	SE	T-value
Older age group	290	0.108^***^	0.006	16.5
Younger age group	1212	0.105^***^	0.005	19.6

To further examine gender heterogeneity in the effects of participation in rural revitalization talent training on social capital growth, the sample is divided into a male group and a female group, and heterogeneous patterns of social capital improvement after training are analyzed across genders. Table 6 reports the corresponding estimation results. The results indicate that participation in rural revitalization talent training has a significantly positive effect on social capital for both male and female farmers, with estimates significant at the 1% level. Specifically, relative to non-participants, the probability of improved social capital increases by approximately 5.1% for male farmers after training, whereas the corresponding increase is about 12.8% for female farmers. This suggests that rural revitalization talent training effectively promotes social capital accumulation for both genders, but the magnitude of the effect differs substantially. In particular, female farmers experience significantly larger gains in social capital than male farmers, consistent with related findings that education and training often yield stronger marginal effects for groups whose pre-existing social networks are relatively constrained, helping them expand social connections and enhance their capacity for social participation [38].

**Table 6:** Comparison of the social capital effects of participation in rural revitalization talent training across gender groups.

	Sample size	ATT	SE	T-value
Male	1088	0.051^***^	0.003	18.9
Female	414	0.128^***^	0.005	24.5

### Robustness tests

To further verify the effect of farmers’ participation in rural revitalization talent training on the enhancement of their social capital, this study conducts robustness checks in the following three aspects.

### Alternative model specification

To further examine whether the main findings are sensitive to alternative model specifications, this study employs a recursive bivariate probit model as a robustness check. Similar to the ESP model, this approach jointly estimates the participation and outcome equations while allowing the error terms of the two equations to be correlated, thereby accounting for potential selection on unobservables.

Table 7 reports the estimation results. The average structural discrete change associated with participation in rural revitalization talent training is 0.0708 (robust SE = 0.0426; z = 1.66; p = 0.097; 95% CI: −0.0128 to 0.1543). This estimate is close to the baseline ESP estimate of 0.068, indicating that the positive association between participation in talent training and farmers’ bonding social capital is stable in terms of direction and magnitude across alternative model specifications. However, the alternative-model estimate is only marginally significant at the 10% level, suggesting that statistical precision is somewhat weaker under this specification.

**Table 7:** Robustness check using a recursive bivariate probit model.

Variable	Estimate	Robust SE	z/χ²	p-value	95% CI
Participation in rural revitalization talent training (average structural discrete change)	0.071	0.043	1.66	0.097	[-0.013, 0.154]
Policy understanding (selection equation)	0.564^***^	0.064	8.78	<0.001	[0.438, 0.689]
Error correlation coefficient ((\rho))	0.233	0.114	χ²=3.86	0.049	[0.001, 0.441]

Notes: The average structural discrete change is calculated after estimating the recursive bivariate probit model. Robust standard errors are reported.

In addition, the coefficient of policy understanding in the participation equation remains positive and statistically significant (coefficient = 0.5635, p < 0.001), and the estimated error correlation coefficient is positive and significant (ρ=0.2325, Wald test p = 0.049). These results further support the need to account for endogenous selection in estimating the effect of training participation. However, this robustness check does not independently verify the exclusion restriction of the instrumental variable.

#### Excluding extreme cropland-area observations.

Given the potential influence of extreme cropland-area observations, this study re-estimates the ESP model after excluding farmers whose cropland area exceeds the 95th and 90th percentiles of the sample distribution. The corresponding cutoff values are 20 mu and 11 mu, respectively.

Table 8 reports the results. After excluding observations above the 95th percentile, the ATT estimate for participation in rural revitalization talent cultivation is 0.092, which remains statistically significant at the 1% level. When observations above the 90th percentile are excluded, the corresponding ATT estimate is 0.090, also significant at the 1% level.

**Table 8:** Robustness check excluding extreme cropland-area observations.

Estimation sample	Sample size	ATT	SE	T-value
Excluding cropland area above P95 (>20 mu)	1,404	0.092^***^	0.003	33.932
Excluding cropland area above P90 (>11 mu)	1,329	0.090^***^	0.003	29.960

These findings indicate that the positive effect of rural revitalization talent training on farmers’ bonding social capital is not driven by extreme cropland-area observations, confirming the robustness of the baseline results.

### Placebo test

**Fig 1:**
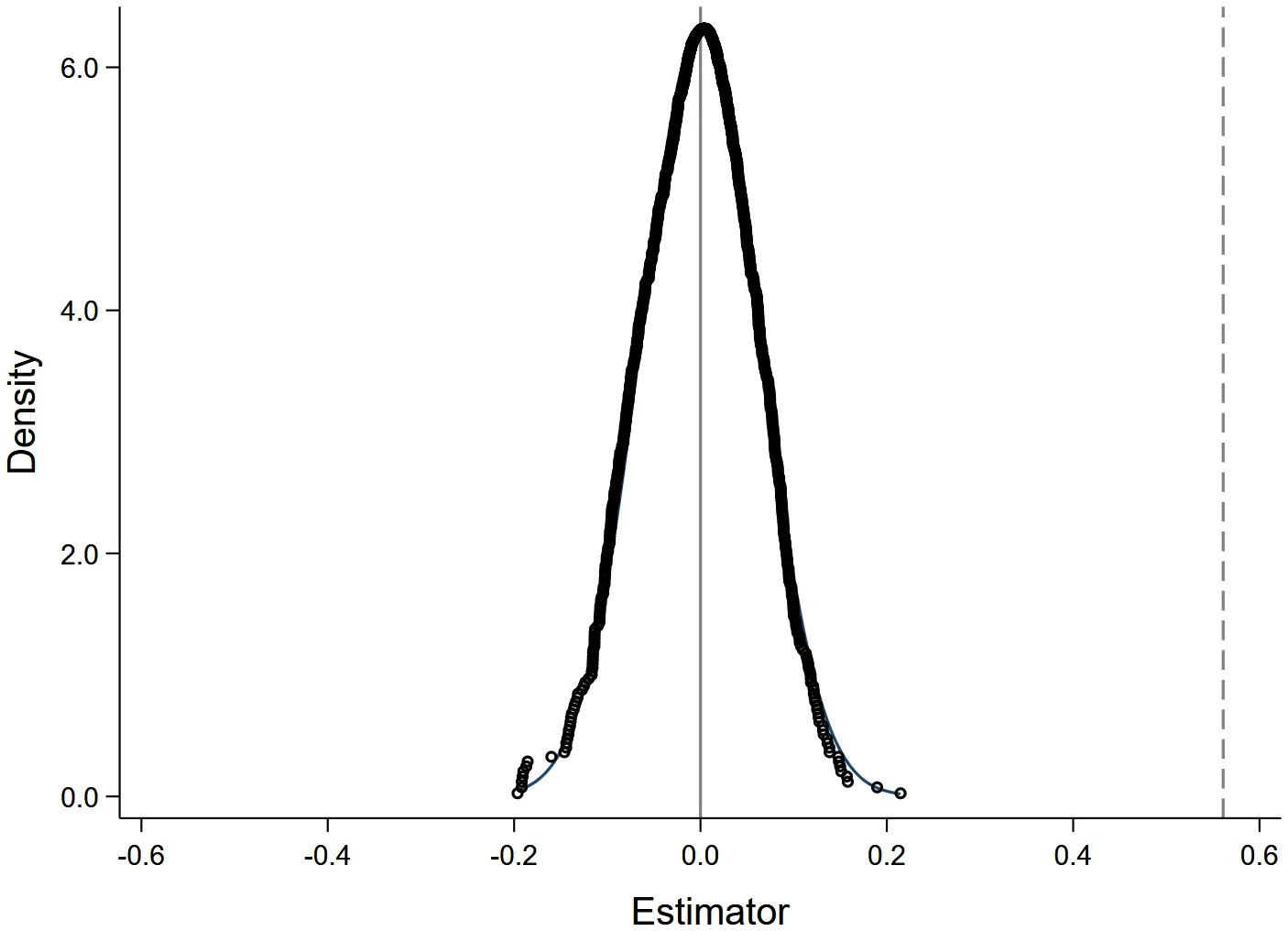
Placebo test.

To assess robustness, this study further conducts a placebo test. By randomly generating a treatment variable and repeatedly re-estimating the model, we construct the distribution of the estimator under a scenario with no true policy shock, as shown in Fig 1. The results indicate that the placebo estimates mostly fluctuate around zero and do not exhibit systematic deviation. In contrast, the baseline estimate lies clearly outside the placebo distribution, suggesting that the identified effect is unlikely to be driven by random factors or model-specification bias. This provides additional evidence that participation in rural revitalization talent training increases farmers’ social capital.

## Discussion

This study systematically examines the impact of rural revitalization talent training on farmers’ social capital and analyzes heterogeneity across dimensions such as training modality and age structure. The results indicate that farmers’ participation in the One Village, One University Student (OVOUS) project significantly increases their level of social capital, and that correspondence education exerts a more pronounced promoting effect than distance education. These findings suggest that institutionalized talent training not only improves farmers’ knowledge and skills, but also—by strengthening interaction intensity and deepening social embeddedness during the learning process—facilitates the expansion of social networks, thereby enhancing farmers’ capacity to access social resources.

In terms of the existing literature, the overall direction of our findings is consistent with prior research on rural education, adult training, and social networks. Related studies generally argue that education and training contribute positively to social capital accumulation by enhancing social participation, expanding the scope of social interactions, and increasing levels of trust [39]. This study further confirms this view within the policy context of rural revitalization and provides contextualized empirical evidence. Compared with studies focusing on years of schooling or general skills training, our analysis centers on a policy-oriented talent-training program with a relatively high degree of institutional embeddedness. Participation in such programs is more strongly selective, which may render training effects more context dependent. We also find substantial differences in social capital gains across age groups, with older farmers experiencing larger improvements than younger farmers. This suggests that, relative to younger farmers whose social networks may already be more mature and whose external connections may be broader, older farmers are more likely to expand their social networks through newly available learning platforms and interaction opportunities after participating in institutionalized training, thereby achieving greater marginal improvements in social capital. Regarding effect sizes, our estimates are more conservative than those reported in some prior studies. This discrepancy is likely attributable to differences in methodology and identification strategies. By using an endogenous switching probit (ESP) model, this study corrects for self-selection into training, whereas some previous studies rely primarily on correlational analysis or control only for observable characteristics, which may overestimate the impact of education on social capital to some extent. Accordingly, our findings can be viewed as complementary evidence on the relationship between rural education and social networks under a stricter identification framework and a clearly specified policy setting.

Nevertheless, this study has several limitations. First, the analysis is based on a sample of farmers from Jiangxi Province, and the external validity of the conclusions should therefore be interpreted with caution. Second, although the ESP model mitigates self-selection bias, it cannot completely eliminate all potential sources of omitted-variable bias. Specifically, unobserved factors that affect farmers’ social capital but are unrelated to the selection process may still influence the estimated effects. Similarly, the PSM estimates rely on the conditional independence assumption and should be interpreted as complementary evidence rather than causal estimates free from all selection concerns. Third, survey-based measures may be subject to social desirability bias. With respect to measurement, due to data constraints, this study uses “whether the farmer can borrow RMB 5,000 from relatives or friends” to proxy bonding social capital. While this indicator captures the capacity to mobilize resources through kinship- and locality-based ties in rural contexts, it cannot fully reflect multidimensional features such as social participation, normative identification, and cross-group ties, and it is particularly limited in capturing bridging social capital. Moreover, if the measurement error in this indicator is classical, the estimated effects may be attenuated toward zero, implying that our estimates may represent a conservative lower bound of the true effect. Future research, where data permit, could incorporate a richer set of indicators to measure different types of social capital more systematically. In addition, cross-regional comparisons and longitudinal follow-up surveys could be used to further test the long-term effects of talent training on social capital and its applicability across different regional contexts. Future work could also integrate perspectives from social psychology to explore the roles of individual traits—such as self-efficacy and risk preferences—in the mechanisms linking training participation to social capital accumulation.

Furthermore, the rapid development of artificial intelligence (AI) and intelligent technologies provides new opportunities for extending research on rural talent cultivation and social capital formation. Recent studies have highlighted the potential of AI-enabled systems in improving personalized learning, optimizing information access, and facilitating intelligent decision-making across different application scenarios [40–42]. Related research also shows that educational and social interventions can significantly shape agricultural engagement and farmers’ behavior. Practical education can strengthen agricultural entrepreneurship intentions by improving individuals’ perceptions of economic benefits, social status, and professional value [43], while ecological education can promote environmentally sustainable behavior [44]. At the farmer level, external supervision and socially embedded factors such as face consciousness also play important roles in shaping agricultural production behavior [45]. These findings highlight the importance of considering both educational interventions and the broader social environment when examining rural human capital development.

Building on these advances, future research could investigate how AI-based educational platforms and intelligent learning tools reshape rural training systems by providing adaptive learning content, reducing geographical barriers, and improving training efficiency. Moreover, AI-driven digital platforms may facilitate farmers’ access to information, market opportunities, and social networks, thereby influencing the formation and transformation of both bonding and bridging social capital. Future studies could further examine the heterogeneous effects of AI-enabled rural education across regions, age groups, and digital capability levels, and explore whether AI technologies can complement practical education and traditional talent-training programs to promote more inclusive and sustainable rural development.

## Conclusions and policy implications

The rural revitalization talent cultivation is crucial to the enhancement of farmers’ social capital, which is not only the inevitable trend of the development of new agricultural management subjects but also the key to realizing the organic connection between small farmers and modern agriculture. Taking the OVOUS project as an example, this study empirically analyses the impact of rural revitalization talent cultivation on farmers’ social capital based on 2,552 farmers’ data, using the endogenous transformation probit model and fully considering the selectivity bias owing to observable and unobservable factors. The results of the study found the following. (1) Generally, farmers’ participation in rural revitalization talent cultivation can significantly enhance their social capital. (2) There are large differences in the impact effects of different types of training on the enhancement of farmers’ social capital. Correspondence education can enhance farmers’ social capital by 8.1%, whereas distance education can only enhance farmers’ social capital by 5.8%. This indicates that the effect of participating in correspondence education on farmers’ social capital enhancement is significantly higher than that of participating in distance education. (3) The results of heterogeneity estimation show that the effect of participating in rural revitalization talent cultivation on the enhancement of social capital is larger for older farmers than for younger farmers, and larger for women than for men.

Based on these findings, we propose the following policy implications:

(1) Expand the functional orientation of talent-training policies. Rural revitalization talent-training policies should not focus solely on improving individual human capital, but should also recognize their potential to strengthen social capital. Our results show that training significantly promotes farmers’ bonding social capital accumulation, thereby exerting positive influences on rural social structures. We recommend that the central government incorporate appropriate social-level indicators into performance evaluation frameworks to more comprehensively capture policy effectiveness.
(2) Optimize training modalities to strengthen social effects. The empirical results indicate that correspondence education is more effective than distance education in promoting social capital, highlighting the importance of interaction frequency and social intensity during the learning process. However, the 2.3-percentage-point advantage of correspondence education over distance education should be considered together with its potentially higher per-participant delivery costs. While expanding remote training, policymakers should pay attention to practical challenges such as resource provision and disparities in digital access, especially in less-developed areas. Local governments can strengthen interaction through measures such as periodic intensive in-person sessions and small-group exchanges, thereby increasing the social-capital returns to training while balancing effectiveness and implementation costs.
(3) Promote differentiated program design. The finding that older farmers experience larger gains in social capital after training indicates that the social effects of educational interventions are heterogeneous across groups. We suggest that local governments adjust course content and organizational arrangements in light of local demographic structures and needs to achieve more precise policy matching.
(4) Improve the accessibility and transparency of policy information. The results show that farmers’ awareness of training policies is significantly associated with their willingness to participate and subsequent social capital outcomes. Although this study does not directly evaluate the causal effects of information campaigns, improving policy awareness may help reduce information barriers and encourage potential participants to access training opportunities. Local governments should strengthen outreach and communication channels, simplify participation procedures, reduce information barriers, and expand policy coverage, thereby enhancing the broader social benefits of these policies.

Overall, rural revitalization talent-training policies are not only an important means of improving individual capabilities, but may also strengthen the social foundations of rural revitalization by reshaping social networks. Future policy design should involve the central government coordinating goal orientation and evaluation standards, while local governments implement programs in a context-sensitive manner. Together, these efforts can jointly promote improvements in both human capital and social capital and fully unlock the potential of such policies.
